# Supplementary material for: Analysis of PPARγ Signaling Activity in Psoriasis
Source: Int J Mol Sci. 2021 Aug 10;22(16):8603. doi: 10.3390/ijms22168603 (PMC8395241; doi:10.3390/ijms22168603)
Supplement: Supplementary file 1 [file ijms-22-08603-s001.zip › Supplemental materials_Analysis of PPARg signaling activity in psoriasis/Pathway models/Models images and html files/Interleukin-17 and interleukin-22 signaling in psoriasis/4524.html]

FOS


# Protein FOS

|  |  |
| --- | --- |
| URN | urn:agi-llid:2353 |
| Total Entities | 0 |
| Connectivity | 7747 |
| Name | FOS |
| Description | FBJ murine osteosarcoma viral oncogene homolog |
| Notes | The Fos gene family consists of 4 members: FOS, FOSB, FOSL1, and FOSL2. These genes encode leucine zipper proteins that can dimerize with proteins of the JUN family, thereby forming the transcription factor complex AP-1. As such, the FOS proteins have been implicated as regulators of cell proliferation, differentiation, and transformation. In some cases, expression of the FOS gene has also been associated with apoptotic cell death. [provided by RefSeq, Jul 2008] |
| Primary Cell Localization | Nucleus |
| Class | Transcription factor |
| ObjectType | Protein |

---

|  |  |
| --- | --- |
| Pathway | Osteoarthritis |
|  | Skin Aging |
|  | Oncogene Induced Cellular Senescence |
|  | Melatonin Effects on Circadian Cycle |
|  | Leptin and CCK8 Activate Nodose Ganglia Neuron |
|  | N-cell: Neurotensin Production |
|  | Dopamine Receptors Signaling |
|  | Prolactin Production |
|  | Thrombopoietin Receptors Signaling in Platelet Maturation |
|  | Cortisol in Resolving Inflammation |
|  | T-Cell Dependent B-Cell Activation |
|  | T-Cell Independent B-Cell Activation |
|  | Natural Killer Cell Activation through C-Type Lectin-like Receptors |
|  | Natural Killer Cell Activation through ITAM-Containing Receptors |
|  | Natural Killer Cell Activation through ITSM-Containing Receptors |
|  | Natural Killer Cell Activation |
|  | CD8+ T-Cell Activation |
|  | Plasmin Effects in Inflammation |
|  | Toll-like Receptor Independent Sterile Inflammation |
|  | Lipoxin A4/FPR2-Related Neutrophil Depression |
|  | Hypothalamic-Pituitary-Adrenal Axis in Acute Phase Response |
|  | Neutrophil Activation via LTB4/Polyisoprenyl Phosphate Signaling |
|  | Mast-Cell Activation without Degranulation through CXCR4 Signaling |
|  | Mast-Cell Activation without Degranulation |
|  | CD16/CD14 Proinflammatory Monocyte Activation |
|  | anti-Inflammatory Function of Macrophage M2 Lineage |
|  | AXL Receptor Inhibits Macrophages and Dendritic Cells Function |
|  | Neutrophil Recruitment and Priming |
|  | Vascular Smooth Muscle Cell/Pericyte Migration and Proliferation |
|  | Vascular Endothelial Cell Activation by Blood Coagulation Factors |
|  | Adipokines Production by Adipocyte |
|  | Nociception Expression Targets Signaling |
|  | NTSR1 Expression Targets |
|  | ADRA1A -> IL6 Production |
|  | CNR1/2 -> IL1B/2/4/6/10 Production |
|  | DRD2 Expression Targets |
|  | DRD1/5 Expression Targets |
|  | PTGER2/3 -> Inflammation-Related Expression Targets |
|  | IL1B Expression Targets -> Nociception |
|  | Melatonin in Cell Survival and Antioxidant Response |
|  | Cell Cycle Overiew |
|  | G0/G1 Phase Transition |
|  | Proteins Involved in Atherosclerosis |
|  | Dendritic Cells Function in Atherosclerosis |
|  | Proteins Involved in Hypertrophic Cardiomyopathy |
|  | Cardiomyocyte Hypertrophy |
|  | Hypertrophic Cardiomyopathy |
|  | Angiotensin-Aldosterone System Activation Scheme |
|  | Endothelin Signaling in Arterial Hypertension |
|  | Arterial Hypertension |
|  | Endothelial Cell Dysfunction in Arterial Hypertension |
|  | Aldosterone Synthesis Chronic Regulation |
|  | Smooth Muscle Cell Dysfunction in Arterial Hypertension |
|  | TRPM4/6/7/8 Signaling in Arterial Hypertension (Hypothesis) |
|  | Myocardial Remodeling |
|  | Proteins Involved in Myocardial Ischemia |
|  | Sebocyte Proliferation in Acne Vulgaris |
|  | Inflammatory Reaction in Acne Vulgaris |
|  | Proteins Involved in Melanoma |
|  | IL17 Signaling in Psoriasis |
|  | IL22 Induces Keratinocyte Proliferation in Psoriasis |
|  | Th0-Cell Aberrant Activation in Systemic Lupus Erythematosus |
|  | B-Cell Activation in Systemic Lupus Erythematosus |
|  | Acute Cytotoxic CD8+T-Cell Response against Melanocytes |
|  | Humoral Immunity in Vitiligo |
|  | Natural Killer Cell in Diabetes Mellitus Type 1 |
|  | Peripheral Tolerance to Autoantigens Recession in Diabetes Mellitus Type 1 |
|  | Tubular Cell Dysfunction in Progressive Diabetic Nephropathy |
|  | Endothelial Cell Dysfunction in Progressive Diabetic Nephropathy |
|  | Endothelial Cell Dysfunction in Early Diabetic Nephropathy |
|  | Mesangial Cell Dysfunction in Diabetic Nephropathy |
|  | Microangiopathy in Diabetic Neuropathy |
|  | Proliferative Diabetic Retinopathy |
|  | Adiponectin Synthesis Declines in Insulin Resistance |
|  | Hepatic Stellate Cells in non-Alcoholic Fatty Liver Disease |
|  | beta-Cell Destruction through Cytokines in Diabetes Mellitus |
|  | beta-Cell Mass Regulation |
|  | Hypothyrodism Associated Hyperprolactinemia |
|  | DRD2 Pharmacological Inhibition in Hyperprolactinemia |
|  | Ethanol Induced Efects in Hyperprolactinemia |
|  | Thyroid Dysfunction |
|  | GNRH Signaling Impairment in Hypogonadotrophic Hypogonadism |
|  | KISS1 Production Impairment in Hypogonadotropic hypogonadism |
|  | Adipokines Production by Adipocyte Impaired in Obesity |
|  | Prolactinoma Overiew |
|  | CD8+ T-Cell Response in Celiac Disease |
|  | GUCY2C Signaling in Colorectal Cancer |
|  | OipA Signaling in Helicobacter Pylori Infection |
|  | CagA Phosphorylation Dependent Signaling |
|  | Epithelial Cell in the Innate Immune Response in Ulcerative Colitis |
|  | T-Cell Cytotoxic Response in Ulcerative Colitis |
|  | Acute Myeloid Leukemia |
|  | Antiphospholipid Antibodies in Endothelial Cell |
|  | B-Cell Acute Lymphoblastic Leukemia |
|  | B-Cell Chronic Lymphocytic Leukemia |
|  | Chronic Myeloid Leukemia |
|  | Follicular Lymphoma |
|  | Osteoclast Activation in Multiple Myeloma |
|  | IL6/IGF1/VEGFA Signaling in Multiple Myeloma |
|  | Multiple Myeloma |
|  | Antiphospholipid Antibodies and Endothelial Cell Interaction |
|  | T-Cell Acute Lymphoblastic Leukemia |
|  | Proteins Involved in Hepatocellular Carcinoma |
|  | NOTCH Signaling in Hepatocellular Carcinoma |
|  | WNT Signaling in Hepatocellular Carcinoma |
|  | Hepatocellular Carcinoma |
|  | Growth Factor Signaling in Hepatocellular Carcinoma |
|  | Pancreatic Ductal Carcinoma |
|  | Growth Factor Signaling in Pancreatic Neoplasms |
|  | Pancreatic Neuroendocrine Tumors |
|  | TRPM7/8 in Pancreatic Adenocarcinoma |
|  | TGFBR Signaling in Pancreatic Neoplasms |
|  | TSLP Signaling in Bronchial Epithelial Cell in Asthma |
|  | Periostin (POSTN) Production by Airway Epithelium in Asthma |
|  | Airway Smooth Muscle Cell High Rate Proliferation |
|  | Osteoclast Activation in Gout |
|  | TNF and IL1B Induce Metalloproteinase Synthesis in Osteoarthritis |
|  | Proteins Involved in Polycystic Ovary Syndrome |
|  | Ovulation Block |
|  | Polycystic Ovary Syndrome |
|  | FSH Action in Polycystic Ovary Syndrome |
|  | Synovial Fibroblast Activation in Psoriatic Arthritis |
|  | Keratinocyte Activation in Psoriatic Arthritis |
|  | PTPN22 Role in Rheumatoid Arthritis |
|  | Synovial Fibroblast Proliferation in Rheumatoid Arthritis |
|  | Osteoclast Activation in Rheumatoid Arthritis |
|  | Synovial Fibroblast Activation by Citokines in Rheumatoid Arthritis |
|  | TLR2 Induced Synovial Fibroblast Activation in Rheumatoid Arthritis |
|  | Autocrine Cytokine/Chemokine Loops in Systemic Scleroderma |
|  | B-Cells Function in Systemic Scleroderma |
|  | Skin Fibrosis |
|  | CCR5 Signaling in Macrophages |
|  | CD8+ T-Cell Action Impairment in HIV Type 1 Infection |
|  | Diabetes Induced Periodontitis |
|  | Estrogen Effects on Pregnancy Gingivitis |
|  | Majeed Syndrome |
|  | Hereditary Breast and Ovarian Cancer Syndrome |
|  | Cowden Syndrome |
|  | Oxytocin Signaling in Brain Nerve Cell (Hypothesis) |
|  | Gluten Impact on Neuronal System (Hypothesis) |
|  | beta-Casomorphins/Opioid Receptors Affect Brain Function (Hypothesis) |
|  | IL6 Promotes Inflammation in Muscular Dystrophies |
|  | Muscular Dystrophies |
|  | IGF1 Role in Muscle Hypertrophy |
|  | Proteins Involved in Osteopetrosis |
|  | Osteoblast Function Decline in Osteoporosis |
|  | Osteoclast Activation in Postmenopause |
|  | Metals and Amyloid beta Toxicity in Alzheimer's Disease |
|  | Proteins Involved in Epilepsy |
|  | Apoptosis and Immediate Early Gene Activation in Epileptiform Disorders |
|  | Glioma Invasion Signaling |
|  | Microglia and Motor Neuron Interaction Dysregulation |
|  | Proteins Involved in Breast Cancer Related to WNT Signaling Pathway |
|  | Proteins Involved in Breast Cancer Related to ESR1 Signaling Pathway |
|  | Proteins Involved in Breast Cancer Related to NOTCH Signaling Pathway |
|  | WNT Signaling in Breast Cancer |
|  | Proteins Involved in Breast Cancer Related to ERBB2/VEGFR/Akt Signaling Pathway |
|  | ERBB/VEGFR/Akt Signaling in Breast Cancer |
|  | IGF1R/AKT Signaling in Breast Cancer |
|  | ESR1/ERBB Positive Luminal Breast Cancer |
|  | Proteins Involved in Breast Cancer Related to IGF1R/Akt Signaling Pathway |
|  | Breast Cancer |
|  | ESR1 Signaling in Breast Cancer |
|  | NOTCH1 Signaling in Breast Cancer |
|  | Endometrial Cancer |
|  | Endometrioid Endometrial Cancer |
|  | Clear Cell Endometrial Cancer and Papillary Serous Endometrial Cancer |
|  | Proteins Involved in Endometriosis |
|  | Angiogenesis in Endometriosis |
|  | Coagulation Factors and PAR1/2 Receptors (F2R/F2RL1) in Endometriosis |
|  | Leptin Role in Endometriosis |
|  | Proteins Involved in High-Grade Serous Ovarian Carcinoma |
|  | Endometrioid Ovarian Carcinoma |
|  | Proteins Involved in Endometrioid Ovarian Carcinoma |
|  | Clear Cell Ovarian Carcinoma |
|  | Proteins Involved in Mucinous Ovarian Carcinoma |
|  | High-Grade Serous Ovarian Carcinoma |
|  | Low-Grade Serous Ovarian Carcinoma |
|  | Proteins Involved in Low-Grade Serous Ovarian Carcinoma |
|  | Ovarian Cancer |
|  | Proteins Involved in Clear Cell Ovarian Carcinoma |
|  | Mucinous Ovarian Carcinoma |
|  | Proteins Involved in Cholesteatoma |
|  | Subepithelial Fibroblasts in Middle Ear Cholesteatoma |
|  | HPV Infection and Cancer |
|  | HPV E5 Regulates EDNRA and EGFR Signaling Pathway in Keratinocytes |
|  | TRPM8 Effects in Prostate Cancer (Hypothesis) |
|  | ER Stress in Macular Degeneration |
|  | Steroids Induced Cataract |
|  | VEGFA in Neovascular Glaucoma |
|  | PGF2-alpha Effect on Treatment |
|  | MUC5AC Expression by NTHi and S. Pneumoniae |
|  | Alveolar Macrophage Negative Regulation Declined in COPD |
|  | Alveolar Macrophages Dysfunction in COPD |
|  | VEGFA Low Expression Level in Chronic Bronchitis |
|  | T-Cell Cytotoxic Activation |
|  | Alveolar Epithelial Cell Dysfunction in COPD |
|  | WNT Signaling in Cystic Kidney Disease |
|  | Polycystins Mutation Effects in Cystic Kidney Disease |
|  | Plasmin Role in Glomerulonephritis |
|  | Endothelial Cell Dysfunction in Glomerulonephritis |
|  | Mesangial Cell Dysfunction in Glomerulonephritis |
|  | Interstitial Fibroblasts in Pyelonephritis |
|  | CD38/CD3 -> JUN/FOS/NF-kB Signaling in T-cell Proliferation |
|  | CD38/CD19 -> JUN/FOS/NF-kB Signaling in B-cell Proliferation |
|  | S100B Expression Targets |
|  | ANGPT2 -> STAT Expression Targets |
|  | ANGPT2 -> AP-1/CREBBP/MYC Expression Targets |
|  | AREG -> AP-1 Expression Targets |
|  | Noradrenaline/Gs Expression Targets |
|  | Dopamine/Gs Expression Targets |
|  | Serotonin/Gs Expression Targets |
|  | Noreadrenaline/Gq Expression Targets |
|  | Epinephrine/Gs Expression Targets |
|  | Acetylcholine Expression Targets |
|  | Serotonin/Gq Expression Targets |
|  | Epinephrine/Gq Expression Targets |
|  | Dopamine/Gi Expression Targets |
|  | B-Cell Receptor -> AP-1 Signaling |
|  | BTC -> AP-1/ATF/CREB Expression Targets |
|  | CD19 Expression Targets |
|  | CD81 -> AP-1 Expression Targets |
|  | CD247 Expression Targets |
|  | gamma Globulins Expression Targets |
|  | FCGR3A Expression Targets |
|  | CD72 -> CREB/CREBBP Expression Targets |
|  | CD8 -> ATF/CREB/CREBBP Expression Targets |
|  | CD80 -> NF-kB Expression Targets |
|  | CD86 -> NF-kB Expression Targets |
|  | CTF1 Expression Targets |
|  | Elevated Receptors -> Expression Targets in Myocardium |
|  | CXCL12 Expression Targets |
|  | CSF2 -> STAT Expression Targets |
|  | LIF Expression Targets |
|  | Clozapine Induced Granulocytopenia |
|  | EGF -> CTNN Expression Targets |
|  | EGF -> AP-1/ATF Expression Targets |
|  | EGF -> CREB/CREBBP/ELK/SRF/MYC Expression Targets |
|  | EGF -> MEF/MYOD/NFATC Expression Targets |
|  | EGF -> STAT Expression Targets |
|  | EGFR -> AP-1/ATF2 Signaling |
|  | EGFR -> AP-1/CREB/ELK/SRF/MYC Signaling |
|  | EREG -> AP-1/ATF Expression Targets |
|  | Erythropoietin -> NF-kB Expression Targets |
|  | Erythropoietin -> AP-1/MYC/CREB Expression Targets |
|  | Erythropoietin -> STAT Expression Targets |
|  | Erythropoietin -> ELK/SRF Expression Targets |
|  | NCAM1 Expression Targets |
|  | F2 -> AP-1/CREB/ELK/SRF/SP1 Expression Targets |
|  | F2 -> STAT1/NF-kB Expression Targets |
|  | PLG -> AP-1/CREB/ELK/SRF/SP1 Expression Targets |
|  | PLG -> STAT1/NF-kB Expression Targets |
|  | FGF9 -> AP-1/CREB/MYC Expression Targets |
|  | FGF10 -> AP-1/CREB/CREBBP/MYC Expression Targets |
|  | FGF2 -> STAT Expression Targets |
|  | FGF1 -> AP-1/CREB/ELK/SRF/MYC Expression Targets |
|  | FGF18 -> STAT Expression Targets |
|  | FGF18 -> AP-1/CREB Expression Targets |
|  | FGF1 -> STAT Expression Targets |
|  | FGF4 -> AP-1/MYC Expression Targets |
|  | FGF7 -> AP-1/CREB/CREBBP/MYC Expression Targets |
|  | FGF2 -> AP-1/CREB/CREBBP/ELK/SRF/MYC Expression Targets |
|  | FGFR -> AP-1/CREB/CREBBP/ELK/SRF/MYC Signaling |
|  | GCG Expression Targets |
|  | ADCYAP1 Expression Targets |
|  | FSHR Expression Targets |
|  | VIP Expression Targets |
|  | Elevated Receptors -> Expression Targets in Liver |
|  | GH1/GHR -> NF-kB/ELK/SRF/MYC Expression Targets |
|  | GH1/GHR -> STAT Expression Targets |
|  | GH1/PRLR Expression Targets |
|  | HBEGF -> AP-1/ATF Expression Targets |
|  | HGF -> STAT Expression Targets |
|  | HGF -> AP-1/CREB/ELK/SRF/MYC Expression Targets |
|  | HGFR -> AP-1/CREB/MYC Signaling |
|  | TAM Receptor Signaling Activation in Cancer |
|  | G0/G1 Cell Cycle Phase Transition Activation in Cancer |
|  | RAS/RAF/MAPK Signaling Activation in Cancer |
|  | WNT Canonical Signaling Activation in Cancer |
|  | Asbestosis- and Silicosis-Associated Inflammation Provokes Cancer Development |
|  | RAS/RAF/MAPK Signaling Activation by Blocking of Tumor Suppressors |
|  | WNT Signaling Activation by Blocking of Tumor Suppressors |
|  | TGFB Signaling activation by Blocking of Tumor Suppressors |
|  | VEGFA Dependent Angiogenesis in Cancer |
|  | ANGPT/TEK Stimulates Endothelial Cell Migration in Cancer |
|  | VEGF Independent Angiogenesis in Cancer |
|  | Integrins in Cancer Cell Motility, Invasion and Survival |
|  | Proteins with Altered Expression in Cancer Metastases |
|  | MTOR/TORC in Cancer Cell Motility and Invasion |
|  | Treg Cells Promote Immunosuppression in Cancer Immune Escape |
|  | IGF1 -> MEF/MYOD/MYOG Expression Targets |
|  | IGF2 -> STAT Expression Targets |
|  | IGF1 -> STAT Expression Targets |
|  | IGF1 -> ELK/SRF/HIF1A/MYC/SREBF Expression Targets |
|  | Insulin -> STAT Expression Targets |
|  | Insulin -> CEBPA/CTNNB/FOXA/FOXO Expression Targets |
|  | Insulin -> MEF/MYOD Expression Targets |
|  | Insulin -> ELK/SRF/HIF1A/MYC/SREBF Expression Targets |
|  | CD19 -> JUN/ELK Signaling |
|  | ICAM1 -> AP-1/CREB/ELK/SRF Signaling |
|  | FibronectinR -> AP-1/ELK/SRF/SREBF Signaling |
|  | CTGF -> AP-1/CREB/MYC Expression Targets |
|  | ICAM1 Expression Targets |
|  | Elevated Receptors -> Expression Targets in Skin |
|  | IFNG/IFNR Expression Targets |
|  | IL7 Expression Targets |
|  | IL13 Expression Targets |
|  | IL16 -> AP-1 Expression Targets |
|  | IL3 Expression Targets |
|  | IL6 Expression Targets |
|  | IL2 Expression Targets |
|  | PTPRJ Expression Targets |
|  | Leptin -> STAT Expression Targets |
|  | Leptin -> ELK/SRF Expression Targets |
|  | Prostaglandin F Expression Targets |
|  | Thromboxane A2 Expression Targets |
|  | LPA Expression Targets |
|  | PAF/Gq -> AP-1/ATF1/CREB/ERK/SRF Expression Targets |
|  | Dronabinol/Anandamide Expression Targets |
|  | Morphine Expression Targets |
|  | S1P Expression Targets |
|  | PGE1 Expression Targets |
|  | Proteins/Ions Transporters -> Expression Targets in Lymphoid System and Blood |
|  | GPCRs Family -> Expression Targets in Lymphoid System and Blood |
|  | MTNR1 Signaling |
|  | NRG1 -> EP300/ETS/ETV/SP1 Expression Targets |
|  | NRG1 -> CREB/CREBBP/ELK/SRF/MYC Expression Targets |
|  | NRG1/Catenin Expression Targets |
|  | NRG1 -> STAT Expression Targets |
|  | NRG1 -> AP-1/ATF Expression Targets |
|  | NCAM1 -> Expression Targets in Nerve Tissue |
|  | GPCRs -> Expression Targets in Brain |
|  | Elevated Receptors -> Expression Targets in Nerve Tissue |
|  | NGF -> SMAD3/NF-kB Expression Targets |
|  | NGF -> AP-1/TP53/MYC Expression Targets |
|  | BDNF Expression Targets |
|  | NTF3 Expression Targets |
|  | NTF4 Expression Targets |
|  | Adenosine Expression Targets |
|  | PDGFB -> AP-1/CREB/MYC Expression Target |
|  | PDGFD -> AP-1 Expression Targets |
|  | PDGF -> AP-1/CREB/CREBBP/MYC Expression Targets |
|  | PDGF -> STAT Expression Targets |
|  | PDGFR -> AP-1/MYC Signaling |
|  | AVP/Gs -> CREB/ELK/SRF/AP-1/EGR Expression Targets |
|  | GNRH1 Expression Targets |
|  | NTS Expression Targets |
|  | GAST Expression Targets in GAST Expression Targets |
|  | GNRH2 Expression Targets |
|  | POMC Expression Targets |
|  | CCL11 Expression Targets |
|  | EDN1 Expression Targets |
|  | AGT -> CREB Expression Targets |
|  | NPY Expression Targets |
|  | AVP/Gq -> STAT Expression Targets |
|  | TAC1 Expression Targets |
|  | AVP/Gs -> STAT Expression Targets |
|  | EDN3 Expression Targets |
|  | AGT -> STAT Expression Targets |
|  | AVP/Gq -> CREB/ELK/SRF/AP-1/EGR Expression Targets |
|  | PRL/PRLR Expression Targets |
|  | PRL/GHR -> STAT Expression Targets |
|  | PRL/GHR -> NF/kB/ELK/SRF/MYC Expression Targets |
|  | PDCD1 -> AP-1 Expression Targets |
|  | AngiopoietinR -> AP-1 Signaling |
|  | EphrinB -> MAPK/JUN/FOS Signaling |
|  | NTRK -> AP-1/CREB/ELK/SRF/MYC/SMAD3/TP53 Signaling |
|  | CSF1 -> STAT Expression Targets |
|  | GAS6 -> AP-1/CREB Expression Targets |
|  | FIGF -> AP-1 Expression Target |
|  | KITLG -> STAT Expression Targets |
|  | KITLG -> AP-1/CREB/CREBBP/MYC Expression Targets |
|  | GDNF -> HSF1 Expression Targets |
|  | PAF Expression Targets |
|  | CSF1 -> AP-1/CREB/CREBBP/MYC Expression Targets |
|  | PGF -> AP-1/CREB/CREBBP/MYC Expression Targets |
|  | FLT3LG -> AP-1/CREB/CREBBP Expression Targets |
|  | TGFBR -> AP-1 Signaling |
|  | TGFBR1/AP-1 Signaling |
|  | TNF-alpha/TNFRSF1A Signaling |
|  | Lysophosphatidic Acid/LPARs Signaling |
|  | JNK/MAPK Signaling |
|  | Estrogens/ESR1 Genomic Canonical Signaling |
|  | MERTK Signaling |
|  | TGF-beta Signaling |
|  | AXL Receptor Tyrosine Kinase Signaling |
|  | TNF-alpha/TNFRSF1B Signaling |
|  | VEGF Signaling |
|  | Estrogens/ESR1 non-Genomic Signaling |
|  | EGFR Signaling |
|  | WNT Planar Cell Polarity (PCP) non-Canonical Signaling |
|  | HRAS Signaling |
|  | ERK5/MAPK7 Signaling |
|  | ERK/MAPK Canonical Signaling |
|  | TCR -> STAT Expression Targets |
|  | TCR -> AP-1 Expression Targets |
|  | TGFA -> STAT Expression Targets |
|  | TGFA -> CTNNB/CTNND Expression Targets |
|  | TGFA/AP-1/ATF Expression Targets |
|  | TGFA -> CREB/CREBBP/ELK-SRF/MYC Expression Targets |
|  | TGFB3-TGFBR2 Expression Targets |
|  | TGFB2-TGFBR2 Expression Targets |
|  | TGFB1-TGFBR2 Expression Targets |
|  | TGFB1-TGFBR1/AP-1 Expression Targets |
|  | FASLG Expression Targets |
|  | TNFSF10 Expression Targets |
|  | LTA Expression Targets |
|  | TNFSF14 Expression Targets |
|  | TNF -> AP-1 Expression Targets |
|  | CD72 -> AP-1 Expression Targets |
|  | TNFSF13 Expression Targets |
|  | TNFSF13B Expression Targets |
|  | TNF -> STAT Expression Targets |
|  | EDA Expression Targets |
|  | EctodysplasinR -> AP-1 Signaling |
|  | TNFR -> AP-1/ATF/TP53 Signaling |
|  | NGFR -> AP-1/CEBPB/CREB/ELK/SRF/TP53 Signaling |
|  | TNFRSF1A -> AP-1/ATF/TP53 Signaling |
|  | Thrombopoietin -> AP-1/CREB/CREBBP/MYC Expression Targets |
|  | TLR4 -> AP-1 Expression Targets |
|  | TLR5 Expression Targets |
|  | TLR7 Expression Targets |
|  | IL1B -> NO Expression Targets |
|  | TLR4 -> AP-1/EGR1/HIF1A Expression Targets |
|  | IL1B -> PGE2 Expression Targets |
|  | TLR1 -> 2/6 Expression Targets |
|  | IL1B Expression Targets |
|  | IL1A Expression Targets |
|  | TLR9 Expression Targets |
|  | TLR -> AP-1 Signaling |
|  | PLAU -> STAT1 Expression Targets |
|  | PLAU -> ELK-SRF/AP-1 Expression Targets |
|  | VEGFA -> ATF/CREB/ELK-SRF Expression Targets |
|  | VEGFA -> STAT Expression Targets |
|  | VEGFA -> AP-1/CREBBP/MYC Expression Targets |
|  | VEGFA -> CTNNB/CTNND Expression Targets |
|  | VEGFR -> AP-1/CREB/MYC Signaling |
|  | pathway 3 |
|  | ace2 |
|  | PPARs and anxiety common targets |
|  | Amygdaloid expressed genes |
|  | New Pathway |
|  | Figure 3\_Mixed network of dexamethasone targets |
|  | Figure 3\_Mixed network of dexamethasone targets |
|  | 1\_Inflammation in keratinocytes in the pathology of acne |
|  | 2\_Hyperseborrhea in acne vulgaris |
|  | 2\_Interleukin-17 and interleukin-22 signaling in psoriasis |
|  | 1\_TNF and IL1B provoke ECM degradation in osteoarthritis |
|  | 1\_Osteoclast-mediated bone resorption in postmenopausal women |
|  | 2\_Osteoblast-mediated bone demineralization |
|  | 1\_Osteoclast functional impairment in osteopetrosis |
|  | 1\_Formation of cysts in polycystic kidney disease |
|  | 3\_The role of interstitial fibroblasts in pyelonephritis |
|  | 3\_Mesangial cell dysfunction in glomerulonephritis |
|  | 1\_Local estrogen production stimulates ectopic endometrium proliferation |
|  | 3\_Enhanced angiogenesis in endometriosis |
|  | 1\_Disruption of luteinizing hormone and follicle-stimulating hormone secretion in PCOS |
|  | 3\_Endoplasmic reticulum stress in age-related macular degeneration |
|  | 2\_Pathogens stimulate mucins expression in the middle ear |
|  | 2\_Cardiomyocyte hypertrophy |
|  | 3\_Dysfunction of smooth muscle cells in hypertension |
|  | 1\_Cigarette smoke causes dysfunction and production of inflammatory mediators in alveolar epithelial cells |
|  | 3\_Role of alveolar macrophages in pulmonary tissue destruction in COPD |
|  | 3\_1\_Mucus accumulation in asthma |
|  | 4\_2\_Airway smooth muscle cells proliferation and airway tissue remodeling |
|  | 4\_Mucin hyperproduction in goblet and mucous cells in COPD |
|  | 2\_Intestine epithelial cells drive immunological responses in ulcerative colitis |
|  | 2\_CD8+ T-cell response and mucosal damage in celiac disease |
|  | 3\_Human papilloma virus E4 and E5 proteins in viral reproduction |
|  | 3\_1\_Beta-cell proliferation and compensation: proliferation |
|  | 3\_Islet beta-cell destruction in T1D |
|  | 1\_Microangiopathy in diabetic neuropathy |
|  | drug-target pairs binding |
|  | Interleukin-17 and interleukin-22 signaling in psoriasis |

---

|  |  |
| --- | --- |
| Group | Proteins Involved in Hereditary Breast and Ovarian Cancer Syndrome |
|  | Proteins Involved in Cowden Syndrome |

---

|  |  |
| --- | --- |
| Source | curated |
|  | ResNet |
|  | GO component |

---

|  |  |
| --- | --- |
| MedScan ID | 2353 |

---

|  |  |
| --- | --- |
| LocusLink ID | 2353 |
|  | 14281 |
|  | 314322 |
|  | 60585 |
|  | 24371 |
|  | 280795 |
|  | 394198 |
|  | 114281 |

---

|  |  |
| --- | --- |
| Alias | cellular oncogene fos |
|  | protooncogene Fos |
|  | protooncogene protein c-Fos |
|  | wb-Fos |
|  | transforming protein Fos |
|  | Cellular oncogene C-FOS |
|  | proto-oncogene cFos |
|  | FBJ murine osteosarcoma viral oncogene homolog |
|  | G0S7 |
|  | FOS\_HUMAN |
|  | Fos proto-oncogene, AP-1 transcription factor subunit |
|  | v-Fos FBJ murine osteosarcoma viral oncogene homolog |
|  | Fos-wb |
|  | FBJ osteosarcoma oncogene |
|  | D12Rfj I |
|  | Foswb |
|  | proto-oncogene c-Fos |
|  | G0/G1 switch regulatory protein 7 |
|  | D12Rfj1 |
|  | cFos |
|  | v-Fos oncogene homolog |
|  | oncogene Fos |
|  | wbFos |
|  | c-fos |
|  | OTTMUSP00000021331 |
|  | v-Fos oncogene |
|  | v-Fos |
|  | Fos proto-oncogene, AP-1 trancription factor subunit |
|  | FBJ murine osteosarcoma viral (v-fos) oncogene homolog (oncogene FOS) |
|  | G0S7 proteins |
|  | FBJ murine osteosarcoma viral (v-fos) oncogene homolog |
|  | FBJ murine osteosarcoma viral |
|  | P55-C-FOS proto-oncogene protein |
|  | Fos |
|  | proto-oncogene Fos |
|  | transcription factor c-Fos |
|  | c-fos oncogene |
|  | LOC196935 |
|  | p55 |
|  | AP-1 |
|  | activator protein 1 |
|  | proto-oncogene protein c-fos |
|  | G0S7 proteins |
|  | G0S7 protein |
|  | wb-fos protein gene |
|  | Proto-oncogene protein c-fos (Cellular oncogene fos) |
|  | v-fos) oncogene homolog (oncogene FOS |
|  | similar to Proto-oncogene protein c-fos (Cellular oncogene fos) |
|  | Manh14 |
|  | mandible shape 14 |
|  | Manh14s |
|  | QTL-SH10.2 |
|  | CH211-153J24.1 |
|  | cb1065 |
|  | chromosome |
|  | scaffold |
|  | zgc:77885 |

---

|  |  |
| --- | --- |
| GO ID | 0001228 |
|  | 0003700 |
|  | 0000981 |
|  | 0070412 |
|  | 0001102 |
|  | 0000979 |
|  | 0000978 |
|  | 0003682 |
|  | 0046982 |
|  | 0044212 |
|  | 0006306 |
|  | 0038095 |
|  | 0060395 |
|  | 0007568 |
|  | 0071276 |
|  | 0071277 |
|  | 0031668 |
|  | 0032870 |
|  | 0034614 |
|  | 0001661 |
|  | 0019221 |
|  | 0007565 |
|  | 0006954 |
|  | 0007399 |
|  | 1901216 |
|  | 0045672 |
|  | 1902895 |
|  | 0045944 |
|  | 0045893 |
|  | 0051090 |
|  | 0006357 |
|  | 0051591 |
|  | 0009409 |
|  | 0051412 |
|  | 0009629 |
|  | 0035902 |
|  | 0009416 |
|  | 0032496 |
|  | 0035994 |
|  | 0032570 |
|  | 0009636 |
|  | 0035914 |
|  | 0030431 |
|  | 0006366 |
|  | 0007179 |
|  | 0005829 |
|  | 0005783 |
|  | 0016020 |
|  | 0043005 |
|  | 0005654 |
|  | 0005634 |
|  | 0032993 |
|  | 0035976 |
|  | 0003677 |
|  | 0003690 |
|  | 0043565 |
|  | 0008134 |
|  | 0010468 |
|  | 0006355 |
|  | 0042493 |
|  | 0005737 |
|  | 0005667 |
|  | 0034097 |
|  | 0009612 |
|  | 0014070 |
|  | 0001077 |
|  | 0001190 |
|  | 0000982 |
|  | 0002755 |
|  | 0002756 |
|  | 0035666 |
|  | 0045087 |
|  | 0051403 |
|  | 0034166 |
|  | 0034134 |
|  | 0034138 |
|  | 0034142 |
|  | 0034146 |
|  | 0034162 |
|  | 0038123 |
|  | 0038124 |
|  | 0002224 |
|  | 0005730 |
|  | 0008063 |
|  | 0034130 |
|  | 0006950 |
|  | 0010843 |
|  | 0003704 |
|  | 0051789 |
|  | 0006351 |
|  | 0019717 |
|  | 0005624 |
|  | 0046332 |
|  | 0046983 |
|  | 0045941 |
|  | 0009987 |
|  | 0051726 |
|  | 0000074 |
|  | 0050875 |
|  | 0005622 |
|  | 0007048 |
|  | 0008151 |
|  | 0005515 |

---

|  |  |
| --- | --- |
| KEGG ID | hsa:2353 |
|  | mmu:14281 |
|  | rno:314322 |

---

|  |  |
| --- | --- |
| MedLine Reference | 10488148 |
|  | 10497212 |
|  | 10523647 |
|  | 10764760 |
|  | 10777532 |
|  | 10799874 |
|  | 10918580 |
|  | 11053448 |
|  | 11134330 |
|  | 11260070 |
|  | 11278640 |
|  | 11397794 |
|  | 11397816 |
|  | 11641401 |
|  | 11846562 |
|  | 11891317 |
|  | 11909859 |
|  | 11983170 |
|  | 12052862 |
|  | 12082260 |
|  | 12127989 |
|  | 12147741 |
|  | 12183893 |
|  | 12193410 |
|  | 12445807 |
|  | 12446585 |
|  | 12477932 |
|  | 12547826 |
|  | 12592382 |
|  | 12600988 |
|  | 12687275 |
|  | 12692226 |
|  | 12705898 |
|  | 12788789 |
|  | 12949493 |
|  | 12972619 |
|  | 1406655 |
|  | 14511403 |
|  | 14517309 |
|  | 14522952 |
|  | 14629301 |
|  | 14702039 |
|  | 14741347 |
|  | 14966901 |
|  | 15188457 |
|  | 15262961 |
|  | 1545828 |
|  | 15507668 |
|  | 15530430 |
|  | 15548518 |
|  | 15588513 |
|  | 15654976 |
|  | 15708845 |
|  | 15767669 |
|  | 1589769 |
|  | 15921770 |
|  | 15926923 |
|  | 16038047 |
|  | 16049073 |
|  | 16055710 |
|  | 16123044 |
|  | 16219292 |
|  | 16236267 |
|  | 1631061 |
|  | 16552729 |
|  | 16637060 |
|  | 16828227 |
|  | 17018293 |
|  | 17182779 |
|  | 1828107 |
|  | 1903538 |
|  | 2414012 |
|  | 3027573 |
|  | 3555978 |
|  | 3817017 |
|  | 6574479 |
|  | 7540136 |
|  | 7665092 |
|  | 7685215 |
|  | 7713192 |
|  | 7816143 |
|  | 7816602 |
|  | 7962462 |
|  | 7994752 |
|  | 8065335 |
|  | 8108109 |
|  | 8248197 |
|  | 8397339 |
|  | 8475068 |
|  | 8557686 |
|  | 8570175 |
|  | 8628277 |
|  | 8710853 |
|  | 8737385 |
|  | 8763994 |
|  | 8904195 |
|  | 8971979 |
|  | 9057648 |
|  | 9121455 |
|  | 9129988 |
|  | 9207128 |
|  | 9334186 |
|  | 9346889 |
|  | 9443941 |
|  | 9468519 |
|  | 9510247 |
|  | 9642216 |
|  | 9671405 |
|  | 9681824 |
|  | 9685505 |
|  | 9732876 |
|  | 9792677 |
|  | 9888853 |
|  | 9918847 |
|  | 10349636 |
|  | 10352021 |
|  | 10524622 |
|  | 10528124 |
|  | 10559418 |
|  | 10569807 |
|  | 10627584 |
|  | 10655067 |
|  | 10711713 |
|  | 10828017 |
|  | 10973734 |
|  | 11039731 |
|  | 11042159 |
|  | 11069886 |
|  | 11076861 |
|  | 11151091 |
|  | 11217851 |
|  | 11566878 |
|  | 11731234 |
|  | 11747369 |
|  | 11755686 |
|  | 11813242 |
|  | 11829488 |
|  | 11925568 |
|  | 11948401 |
|  | 11961557 |
|  | 12054176 |
|  | 12082614 |
|  | 12083820 |
|  | 12091449 |
|  | 12219089 |
|  | 12220541 |
|  | 12239115 |
|  | 12366817 |
|  | 12376177 |
|  | 12383919 |
|  | 12438440 |
|  | 12466851 |
|  | 12486129 |
|  | 12489182 |
|  | 12562866 |
|  | 12595280 |
|  | 12654520 |
|  | 12694872 |
|  | 12719996 |
|  | 12782152 |
|  | 12876470 |
|  | 12882314 |
|  | 12902987 |
|  | 12943993 |
|  | 1329091 |
|  | 1383034 |
|  | 1406937 |
|  | 1423615 |
|  | 14511666 |
|  | 14526314 |
|  | 14534245 |
|  | 14555986 |
|  | 1465144 |
|  | 14656296 |
|  | 14699150 |
|  | 14706332 |
|  | 14706339 |
|  | 14980515 |
|  | 14982872 |
|  | 15005710 |
|  | 15056714 |
|  | 15067049 |
|  | 15073183 |
|  | 1508191 |
|  | 15121008 |
|  | 15143165 |
|  | 15169892 |
|  | 1517237 |
|  | 15182309 |
|  | 15207916 |
|  | 15247511 |
|  | 15292661 |
|  | 15294973 |
|  | 15464763 |
|  | 15476581 |
|  | 15519672 |
|  | 15522236 |
|  | 15590632 |
|  | 15607821 |
|  | 15618518 |
|  | 15619674 |
|  | 15719069 |
|  | 15721299 |
|  | 15733037 |
|  | 15752732 |
|  | 15758572 |
|  | 15772364 |
|  | 15795322 |
|  | 15834895 |
|  | 15913695 |
|  | 15944271 |
|  | 1594760 |
|  | 15972968 |
|  | 16002216 |
|  | 16007083 |
|  | 1605850 |
|  | 16141072 |
|  | 16141073 |
|  | 1616905 |
|  | 16172035 |
|  | 16263220 |
|  | 16288200 |
|  | 16313514 |
|  | 16359817 |
|  | 16424941 |
|  | 16434964 |
|  | 16530956 |
|  | 16569682 |
|  | 16636292 |
|  | 16687291 |
|  | 16892556 |
|  | 16916723 |
|  | 16966486 |
|  | 17066317 |
|  | 17130154 |
|  | 1889815 |
|  | 1909952 |
|  | 1914521 |
|  | 1957173 |
|  | 1970330 |
|  | 2140597 |
|  | 2351070 |
|  | 2452194 |
|  | 2506639 |
|  | 2515037 |
|  | 2575583 |
|  | 3004967 |
|  | 3082981 |
|  | 3108379 |
|  | 3127059 |
|  | 3147218 |
|  | 3186715 |
|  | 3457564 |
|  | 3894012 |
|  | 6088665 |
|  | 6090941 |
|  | 6093261 |
|  | 6205276 |
|  | 6289129 |
|  | 6292525 |
|  | 6301687 |
|  | 6306448 |
|  | 6319013 |
|  | 6479151 |
|  | 6514007 |
|  | 6541297 |
|  | 6607118 |
|  | 6641714 |
|  | 7476995 |
|  | 7514103 |
|  | 7545543 |
|  | 7547979 |
|  | 7595148 |
|  | 7600583 |
|  | 7600678 |
|  | 7601910 |
|  | 7656403 |
|  | 7689683 |
|  | 7700631 |
|  | 7720577 |
|  | 7741720 |
|  | 7758695 |
|  | 7806225 |
|  | 7807002 |
|  | 7824281 |
|  | 7841657 |
|  | 7910456 |
|  | 7911121 |
|  | 7922576 |
|  | 7929053 |
|  | 7939685 |
|  | 7955078 |
|  | 7989745 |
|  | 8007962 |
|  | 8007971 |
|  | 8017775 |
|  | 8025159 |
|  | 8036015 |
|  | 8036026 |
|  | 8039503 |
|  | 8082881 |
|  | 8088780 |
|  | 8095010 |
|  | 8114694 |
|  | 8131748 |
|  | 8134124 |
|  | 8136308 |
|  | 8144618 |
|  | 8150544 |
|  | 8179594 |
|  | 8194766 |
|  | 8196666 |
|  | 8198544 |
|  | 8258699 |
|  | 8264605 |
|  | 8275705 |
|  | 8276400 |
|  | 8281019 |
|  | 8286110 |
|  | 8335693 |
|  | 8364887 |
|  | 8369223 |
|  | 8397429 |
|  | 8405990 |
|  | 8479285 |
|  | 8482578 |
|  | 8486351 |
|  | 8491516 |
|  | 8565820 |
|  | 8566348 |
|  | 8573144 |
|  | 8580740 |
|  | 8612296 |
|  | 8643637 |
|  | 8656277 |
|  | 8668344 |
|  | 8678994 |
|  | 8750842 |
|  | 8754282 |
|  | 8757326 |
|  | 8777432 |
|  | 8790912 |
|  | 8793856 |
|  | 8800404 |
|  | 8831695 |
|  | 8847666 |
|  | 8896447 |
|  | 9055866 |
|  | 9058381 |
|  | 9098922 |
|  | 9099259 |
|  | 9120437 |
|  | 9162016 |
|  | 9169087 |
|  | 9180190 |
|  | 9183740 |
|  | 9210523 |
|  | 9221926 |
|  | 9310300 |
|  | 9344655 |
|  | 9664690 |
|  | 9710257 |
|  | 9710644 |
|  | 9723028 |
|  | 9799237 |
|  | 9804131 |
|  | 9840922 |
|  | 9858769 |
|  | 9876179 |
|  | 9918819 |
|  | 11025764 |
|  | 11766894 |
|  | 11790471 |
|  | 11846448 |
|  | 11850142 |
|  | 11861125 |
|  | 11939505 |
|  | 11956153 |
|  | 12064606 |
|  | 12121970 |
|  | 12145054 |
|  | 12184859 |
|  | 12215476 |
|  | 12370534 |
|  | 12384476 |
|  | 12429561 |
|  | 12443984 |
|  | 12528378 |
|  | 12531532 |
|  | 12535784 |
|  | 12606042 |
|  | 12629509 |
|  | 12638111 |
|  | 12714357 |
|  | 12787062 |
|  | 12834907 |
|  | 12946711 |
|  | 12965235 |
|  | 1298921 |
|  | 14576829 |
|  | 15306261 |
|  | 2105492 |
|  | 3140224 |
|  | 3325886 |
|  | 8889548 |
|  | 9434195 |
|  | 10199628 |
|  | 11766904 |
|  | 11861123 |
|  | 12802426 |
|  | 14625026 |
|  | 14674859 |
|  | 14757116 |
|  | 14767061 |
|  | 14979778 |
|  | 15024755 |
|  | 15033433 |
|  | 15044536 |
|  | 15051527 |
|  | 15115808 |
|  | 15136564 |
|  | 15189768 |
|  | 15249992 |
|  | 15254773 |
|  | 15334681 |
|  | 15380004 |
|  | 15453261 |
|  | 15458969 |
|  | 15609068 |
|  | 15701816 |
|  | 15702242 |
|  | 15842237 |
|  | 15922053 |
|  | 15926928 |
|  | 16055265 |
|  | 16095819 |
|  | 16097052 |
|  | 16110275 |
|  | 16130823 |
|  | 16344157 |
|  | 16531639 |
|  | 16547969 |
|  | 16604387 |
|  | 16633904 |
|  | 16926533 |
|  | 17005860 |
|  | 17020011 |
|  | 17101878 |
|  | 17111371 |
|  | 17147208 |
|  | 7990028 |
|  | 8206384 |
|  | 9879931 |
|  | 11282978 |
|  | 16305752 |
|  | 15730879 |
|  | 16000406 |
|  | 16379021 |
|  | 12508121 |
|  | 15489334 |
|  | 1658710 |
|  | 17160021 |
|  | 17203973 |
|  | 17209045 |
|  | 17235455 |
|  | 17606925 |
|  | 2511004 |
|  | 2516827 |
|  | 10820233 |
|  | 11106803 |
|  | 11134582 |
|  | 12766089 |
|  | 15157984 |
|  | 16275763 |
|  | 16339574 |
|  | 17141758 |
|  | 17178788 |
|  | 17189721 |
|  | 17192632 |
|  | 17392792 |
|  | 17458858 |
|  | 17482371 |
|  | 17485464 |
|  | 17531086 |
|  | 2991903 |
|  | 15014501 |
|  | 16962079 |
|  | 17006656 |
|  | 17120244 |
|  | 17152352 |
|  | 17194726 |
|  | 17216194 |
|  | 17218446 |
|  | 17244536 |
|  | 17448703 |
|  | 17514764 |
|  | 17622784 |
|  | 17654838 |
|  | 17661378 |
|  | 9571165 |
|  | 9847304 |

---

|  |  |
| --- | --- |
| Organism | Homo sapiens {Organism urn:agi-taxid:9606} |
|  | Mus musculus {Organism urn:agi-taxid:10090} |
|  | Rattus norvegicus {Organism urn:agi-taxid:10116} |
|  | Homo sapiens |
|  | Mus musculus |
|  | Rattus norvegicus |
|  | Human |
|  | rat |
|  | mouse |
|  | Danio rerio |
|  | Bos taurus |

---

|  |  |
| --- | --- |
| Mouse chromosome position | 12 39.7 cM |
|  | 12 40.0 cM |

---

|  |  |
| --- | --- |
| OMIM ID | 164810 |
|  | 2148821 |
|  | OMIM:164810 |

---

|  |  |
| --- | --- |
| Rat chromosome position | 6q31 |

---

|  |  |
| --- | --- |
| Hugo ID | 3796 |
|  | HGNC:3796 |

---

|  |  |
| --- | --- |
| Human chromosome position | 14q24.3 |

---

|  |  |
| --- | --- |
| Swiss-Prot Accession | P01100 |
|  | Q6FG41 |
|  | P01100.1 |
|  | P01101 |
|  | P01101.1 |
|  | P12841 |
|  | P12841.1 |
|  | A8K4E2 |
|  | B4DQ65 |
|  | P18849 |
|  | Q4FDN1 |
|  | Q76MY9 |
|  | Q76MZ0 |
|  | Q3U463 |
|  | Q6TDG4 |
|  | Q5G6W2 |
|  | Q5G6W3 |
|  | Q5U7E3 |
|  | Q5U873 |
|  | Q5U874 |
|  | Q5U875 |
|  | Q63752 |
|  | Q9EQC2 |
|  | FOS\_HUMAN |
|  | FOS\_MOUSE |
|  | FOS\_RAT |
|  | Q6FG41\_HUMAN |

---

|  |  |
| --- | --- |
| PIR ID | A01342 |
|  | E34223 |
|  | A01343 |
|  | A28263 |

---

|  |  |
| --- | --- |
| GenBank ID | NC\_000014 |
|  | NM\_005252 |
|  | NP\_005243 |
|  | NG\_029673 |
|  | AB022275 |
|  | BAA87920 |
|  | AB022276 |
|  | BAA87921 |
|  | AF111167 |
|  | AAC98315 |
|  | AY212879 |
|  | AAO21129 |
|  | CH471061 |
|  | EAW81227 |
|  | EAW81228 |
|  | EAW81229 |
|  | HI574162 |
|  | CBX47570 |
|  | K00650 |
|  | AAA52471 |
|  | KT583865 |
|  | V01512 |
|  | CAA24756 |
|  | AH003773 |
|  | AAB20306 |
|  | AK097379 |
|  | BAG53458 |
|  | AK290907 |
|  | BAF83596 |
|  | AK291326 |
|  | BAF84015 |
|  | AK298659 |
|  | BAG60827 |
|  | BC004490 |
|  | AAH04490 |
|  | BX647104 |
|  | CR541785 |
|  | CAG46584 |
|  | CR542267 |
|  | CAG47063 |
|  | P01100 |
|  | NC\_000078 |
|  | NM\_010234 |
|  | NP\_034364 |
|  | AC159244 |
|  | CH466590 |
|  | EDL02869 |
|  | EDL02870 |
|  | EDL02871 |
|  | J00370 |
|  | AAA96699 |
|  | M23768 |
|  | V00727 |
|  | CAA24105 |
|  | AK154418 |
|  | BAE32571 |
|  | AK159688 |
|  | AY423854 |
|  | AAR00345 |
|  | BC013242 |
|  | BC029814 |
|  | AAH29814 |
|  | P01101 |
|  | NC\_005105 |
|  | NM\_022197 |
|  | NP\_071533 |
|  | AC\_000074 |
|  | AABR07065091 |
|  | AAHX01045615 |
|  | AF126534 |
|  | AF277645 |
|  | AAG47951 |
|  | AY780201 |
|  | AAV33852 |
|  | AY780202 |
|  | AAV33853 |
|  | AY780203 |
|  | AAV33854 |
|  | AY786174 |
|  | AAV41063 |
|  | AY828998 |
|  | AAW65541 |
|  | AY828999 |
|  | AAW65542 |
|  | CH473982 |
|  | EDL81573 |
|  | EDL81574 |
|  | EDL81575 |
|  | DQ089699 |
|  | AAZ13764 |
|  | M34001 |
|  | AAA42348 |
|  | U02631 |
|  | FQ225996 |
|  | FQ228884 |
|  | X06769 |
|  | CAA29937 |
|  | P12841 |
|  | NC\_018925 |
|  | AC\_000146 |
|  | ABBA01030704 |
|  | AMYH02029536 |
|  | S65138 |
|  | DQ893444 |
|  | ABM84370 |
|  | DQ896750 |
|  | ABM87749 |
|  | AC\_000034 |
|  | AAHY01101991 |
|  | XM\_005267488 |
|  | XP\_005267545 |
|  | AABR06045297 |
|  | NT\_026437 |
|  | NW\_001838113 |
|  | NW\_925561 |
|  | AC\_000057 |
|  | CR612516 |
|  | CR620080 |
|  | Q6FG41 |
|  | Q76MY9 |
|  | Q76MZ0 |
|  | NT\_039551 |
|  | NW\_001030502 |
|  | Q3U463 |
|  | Q6TDG4 |
|  | NW\_001084846 |
|  | NW\_047762 |
|  | Q5G6W2 |
|  | Q5G6W3 |
|  | Q5U7E3 |
|  | Q5U873 |
|  | Q5U874 |
|  | Q5U875 |
|  | Q63752 |
|  | Q9EQC2 |
|  | AI059251 |
|  | NW\_000053 |
|  | AV026617 |
|  | BF547581 |
|  | 119601633 |
|  | 119601634 |
|  | 120470 |
|  | 124000481 |
|  | 13325364 |
|  | 182735 |
|  | 27802689 |
|  | 29904 |
|  | 4063509 |
|  | 49456527 |
|  | 49457528 |
|  | 6518629 |
|  | 6518635 |
|  | 74712937 |
|  | 74712938 |
|  | 74748440 |
|  | 120471 |
|  | 123787535 |
|  | 20988257 |
|  | 37695628 |
|  | 387161 |
|  | 50400 |
|  | 74181717 |
|  | 81864184 |
|  | 207685 |
|  | 120473 |
|  | 12056584 |
|  | 123781712 |
|  | 54398906 |
|  | 54398908 |
|  | 54398910 |
|  | 54873705 |
|  | 55934 |
|  | 58189915 |
|  | 58189917 |
|  | 70888329 |
|  | 81862069 |
|  | 81862070 |
|  | 81862661 |
|  | 81862662 |
|  | 81862663 |
|  | 81871952 |
|  | 81890364 |
|  | 81906232 |
|  | Q4FDN1 |
|  | NT\_086807 |
|  | XP\_234422 |
|  | XM\_234422 |
|  | 109659357 |
|  | 120419460 |
|  | 3212099 |
|  | 32481980 |
|  | 76789652 |
|  | AAC21577 |
|  | AAI18281 |
|  | AAP84343 |
|  | ABM21549 |
|  | AF069515 |
|  | AY322482 |
|  | BC118280 |
|  | BT029837 |
|  | NC\_007308 |
|  | NM\_182786 |
|  | NP\_877587 |
|  | NW\_001492868 |
|  | O77628 |
|  | 123905118 |
|  | 41107629 |
|  | 55962867 |
|  | 55962868 |
|  | 82079034 |
|  | 82202318 |
|  | 87241870 |
|  | AAH65466 |
|  | AAY21256 |
|  | AL929435 |
|  | BC065466 |
|  | CAI11634 |
|  | CAI11635 |
|  | DQ003339 |
|  | NC\_007131 |
|  | NM\_205569 |
|  | NP\_991132 |
|  | NW\_001512152 |
|  | Q4ZJF0 |
|  | Q5SP68 |
|  | Q6P0S4 |
|  | 119601635 |
|  | 123993537 |
|  | 148670922 |
|  | 148670923 |
|  | 148670924 |
|  | 149025206 |
|  | 149025207 |
|  | 149025208 |
|  | 6518634 |
|  | 27802688 |
|  | 34935858 |
|  | 34867433 |
|  | 34867434 |
|  | 4836596 |
|  | 12056583 |
|  | 413826 |
|  | 55933 |
|  | 51766990 |
|  | 31560587 |
|  | 6753894 |
|  | 193342 |
|  | 192565 |
|  | 50399 |
|  | 15301524 |
|  | 20988256 |
|  | 4572570 |
|  | 182734 |
|  | 29903 |
|  | 21757114 |
|  | 33872858 |
|  | 34366132 |
|  | 49456526 |
|  | 49457527 |
|  | 68869 |
|  | 68871 |
|  | NW\_043958 |
|  | 207684 |
|  | 3333028 |
|  | 11638646 |
|  | 51493278 |
|  | 6552332 |
|  | 4885241 |
|  | 51472322 |
|  | 6518628 |
|  | ZDB-GENE-031222-4 |
|  | 45387566 |
|  | 45387567 |
|  | 126007383 |
|  | 394198 |
|  | Dr.12986 |
|  | dre:394198 |

---

|  |  |
| --- | --- |
| Swiss-Prot ID | FOS\_HUMAN |
|  | FOS\_MOUSE |
|  | FOS\_RAT |
|  | Q6FG41\_HUMAN |

---

|  |  |
| --- | --- |
| Cell Localization | Nucleus |
|  | Endoplasmic reticulum |
|  | Cytoplasm |
|  | Cytosol |
|  | Cytoplasm, cytosol |
|  | Intracellular |

---

|  |  |
| --- | --- |
| Ensembl ID | ENSG00000170345 |
|  | ENSP00000306245.4 |
|  | ENST00000303562.9 |
|  | ENSMUSG00000021250 |
|  | ENSMUSP00000021674.6 |
|  | ENSMUST00000021674.6 |
|  | ENSRNOG00000008015 |
|  | ENSRNOP00000010712.2 |
|  | ENSRNOT00000010712.3 |
|  | ENST00000303562.8 |
|  | ENSP00000306245 |
|  | ENST00000303562 |
|  | ENSMUSP00000021674 |
|  | ENSMUST00000021674 |
|  | ENSRNOP00000010712 |
|  | ENSRNOT00000010712 |

---

|  |  |
| --- | --- |
| MGI ID | MGI:95574 |
|  | 95574 |
|  | 2148821 |

---

|  |  |
| --- | --- |
| RGD ID | 2626 |
|  | 735054 |

---

|  |  |
| --- | --- |
| Unigene ID | Rn.103750 |
|  | Hs.25647 |
|  | Mm.246513 |
|  | Hs.728789 |
|  | Hs.718415 |
|  | Hs.568953 |
|  | Bt.52605 |
|  | Dr.12986 |
|  | Mm.5043 |

---

|  |  |
| --- | --- |
| Homologene ID | 3844 |

---

|  |  |
| --- | --- |
| Shape | O-vertex |

---

|  |  |
| --- | --- |
| IPI ID | IPI00131985 |
|  | IPI00033008 |
|  | IPI00368000 |

---

|  |  |
| --- | --- |
| Proteome Biochemical Function | DNA-binding protein |

---

|  |  |
| --- | --- |
| Chromosome position | 12 40.0 cM |
|  | 6q31 |
|  | 14q24.3 |

---

|  |  |
| --- | --- |
| Microarray ID | 160901\_at |
|  | 161716\_at |
|  | 1371283\_at |
|  | x03347cds\_g\_at |
|  | x06769cds\_at |
|  | x06769cds\_g\_at |
|  | A\_42\_P552092 |
|  | 100490\_at |
|  | A\_65\_P03817 |
|  | A\_51\_P333146 |
|  | A\_52\_P262219 |
|  | V01512 |
|  | 229173\_at |
|  | 71524\_at |
|  | 88628\_at |
|  | 209189\_at |
|  | 1915\_s\_at |
|  | 1916\_s\_at |
|  | 2094\_s\_at |
|  | V01512\_rna1\_at |
|  | rc\_n23730\_s\_at |
|  | A\_32\_P75334 |
|  | A\_23\_P106192 |
|  | A\_14\_P107571 |
|  | A\_14\_P117160 |
|  | A\_23\_P106194 |
|  | M494 |
|  | C277 |
|  | OH8074 |
|  | OR3846 |
|  | MB484 |
|  | R549 |
|  | OR0720 |
|  | 203358\_Rn |
|  | 1423100\_at |
|  | 97628\_at |
|  | v00727\_s\_at |
|  | Msa.2013.0\_at |
|  | A\_65\_P07422 |
|  | H3084G03-3 |
|  | NM\_010234.1 |
|  | NM\_010234 |
|  | 234463\_at |
|  | I\_959033 |

---

|  |  |
| --- | --- |
| Homo sapiens Chromosome position | 14q24.3 |

---

|  |  |
| --- | --- |
| Ensembl Transcript ID | ENST00000303562 |

---

|  |  |
| --- | --- |
| ENSEMBL | ENSG00000170345 |

---

|  |  |
| --- | --- |
| Proteome Cellular Role | Chromatin/chromosome structure |
|  | Pol II transcription |

---

|  |  |
| --- | --- |
| FunctionalClass | DNA binding |
|  | double-stranded DNA binding |
|  | protein heterodimerization |
|  | specific RNA polymerase II transcription factor |
|  | transcription factor |
|  | protein dimerization |
|  | sequence-specific DNA binding |
|  | contributes\_to |

---

|  |  |
| --- | --- |
| Molecular Function | Transcription Factor |

---

|  |  |
| --- | --- |
| Hugo Symbol | FOS |

---

|  |  |
| --- | --- |
| Bos taurus Chromosome position | 10 |

---

|  |  |
| --- | --- |
| Mus musculus Chromosome position | 12 40.0 cM |

---

|  |  |
| --- | --- |
| Proteome Subcellular Localization | Nuclear |

---

|  |  |
| --- | --- |
| Compartment | void |
|  | nucleus |

---

|  |  |
| --- | --- |
| KEGG pathway | B cell receptor signaling pathway |
|  | Colorectal cancer |
|  | MAPK signaling pathway |
|  | T cell receptor signaling pathway |
|  | Toll-like receptor signaling pathway |

---

|  |  |
| --- | --- |
| ProteinAtlas SubcellularLocation | Main location: Nucleus but not nucleoli; Other location: ; Expression type: Staining; Reliability: Supportive |

---

|  |  |
| --- | --- |
| RefSeq ACCNUM | NP\_005243 |

---

|  |  |
| --- | --- |
| State | p |

---

|  |  |
| --- | --- |
| Rattus norvegicus Chromosome position | 6q31 |

---

|  |  |
| --- | --- |
| Ensembl Gene ID | ENSG00000170345 |

---

|  |  |
| --- | --- |
| Keyword | transcription factor |
|  | nucleus |
|  | early growth response |
|  | RAS small monomeric GTPase |
|  | oncogene |
|  | leucine zipper |
|  | bzip transcription factor |
|  | hnRNA |
|  | antisense RNA |
|  | cytoplasm |
|  | factor C |
|  | DNA bending |
|  | RNA |
|  | thrombin |
|  | DNA binding activity |
|  | binding activity |
|  | cytosol |
|  | nuclei |
|  | septum |
|  | leucine repeat |
|  | basic leucine zipper |
|  | growth-associated |

---

|  |  |
| --- | --- |
| ProteinAtlas NormalTissue | Tissue: liver; Cell type: hepatocytes; Level: Moderate; Expression type: Staining; Reliability: Supportive |
|  | Tissue: liver; Cell type: bile duct cells; Level: Negative; Expression type: Staining; Reliability: Supportive |
|  | Tissue: breast; Cell type: glandular cells; Level: Weak; Expression type: Staining; Reliability: Supportive |
|  | Tissue: urinary bladder; Cell type: urothelial cells; Level: Strong; Expression type: Staining; Reliability: Supportive |
|  | Tissue: uterus |
|  | post-menopause; Cell type: glandular cells; Level: Moderate; Expression type: Staining; Reliability: Supportive |
|  | Tissue: parathyroid gland; Cell type: glandular cells; Level: Strong; Expression type: Staining; Reliability: Supportive |
|  | Tissue: lymph node; Cell type: germinal center cells; Level: Negative; Expression type: Staining; Reliability: Supportive |
|  | Tissue: bone marrow; Cell type: hematopoietic cells; Level: Moderate; Expression type: Staining; Reliability: Supportive |
|  | Tissue: bronchus; Cell type: respiratory epithelial cells; Level: Strong; Expression type: Staining; Reliability: Supportive |
|  | post-menopause; Cell type: cells in endometrial stroma; Level: Weak; Expression type: Staining; Reliability: Supportive |
|  | Tissue: pancreas; Cell type: islets of Langerhans; Level: Weak; Expression type: Staining; Reliability: Supportive |
|  | Tissue: rectum; Cell type: glandular cells; Level: Moderate; Expression type: Staining; Reliability: Supportive |
|  | Tissue: adrenal gland; Cell type: glandular cells; Level: Strong; Expression type: Staining; Reliability: Supportive |
|  | Tissue: appendix; Cell type: glandular cells; Level: Moderate; Expression type: Staining; Reliability: Supportive |
|  | Tissue: appendix; Cell type: lymphoid tissue; Level: Negative; Expression type: Staining; Reliability: Supportive |
|  | Tissue: cerebellum; Cell type: cells in granular layer; Level: Negative; Expression type: Staining; Reliability: Supportive |
|  | Tissue: cerebellum; Cell type: cells in molecular layer; Level: Negative; Expression type: Staining; Reliability: Supportive |
|  | Tissue: cerebellum; Cell type: Purkinje cells; Level: Moderate; Expression type: Staining; Reliability: Supportive |
|  | Tissue: cerebral cortex; Cell type: glial cells; Level: Weak; Expression type: Staining; Reliability: Supportive |
|  | Tissue: cerebral cortex; Cell type: neuronal cells; Level: Moderate; Expression type: Staining; Reliability: Supportive |
|  | Tissue: cervix, uterine; Cell type: glandular cells; Level: Negative; Expression type: Staining; Reliability: Supportive |
|  | Tissue: cervix, uterine; Cell type: squamous epithelial cells; Level: Strong; Expression type: Staining; Reliability: Supportive |
|  | Tissue: colon; Cell type: glandular cells; Level: Moderate; Expression type: Staining; Reliability: Supportive |
|  | Tissue: duodenum; Cell type: glandular cells; Level: Moderate; Expression type: Staining; Reliability: Supportive |
|  | Tissue: epididymis; Cell type: glandular cells; Level: Weak; Expression type: Staining; Reliability: Supportive |
|  | Tissue: esophagus; Cell type: squamous epithelial cells; Level: Moderate; Expression type: Staining; Reliability: Supportive |
|  | Tissue: fallopian tube; Cell type: glandular cells; Level: Negative; Expression type: Staining; Reliability: Supportive |
|  | Tissue: gall bladder; Cell type: glandular cells; Level: Strong; Expression type: Staining; Reliability: Supportive |
|  | Tissue: heart muscle; Cell type: myocytes; Level: Moderate; Expression type: Staining; Reliability: Supportive |
|  | Tissue: hippocampus; Cell type: glial cells; Level: Negative; Expression type: Staining; Reliability: Supportive |
|  | Tissue: hippocampus; Cell type: neuronal cells; Level: Weak; Expression type: Staining; Reliability: Supportive |
|  | Tissue: kidney; Cell type: cells in glomeruli; Level: Moderate; Expression type: Staining; Reliability: Supportive |
|  | Tissue: kidney; Cell type: cells in tubules; Level: Moderate; Expression type: Staining; Reliability: Supportive |
|  | Tissue: lateral ventricle; Cell type: glial cells; Level: Negative; Expression type: Staining; Reliability: Supportive |
|  | Tissue: lateral ventricle; Cell type: neuronal cells; Level: Weak; Expression type: Staining; Reliability: Supportive |
|  | Tissue: lung; Cell type: macrophages; Level: Weak; Expression type: Staining; Reliability: Supportive |
|  | Tissue: lung; Cell type: pneumocytes; Level: Weak; Expression type: Staining; Reliability: Supportive |
|  | Tissue: lymph node; Cell type: non-germinal center cells; Level: Negative; Expression type: Staining; Reliability: Supportive |
|  | Tissue: nasopharynx; Cell type: respiratory epithelial cells; Level: Moderate; Expression type: Staining; Reliability: Supportive |
|  | Tissue: oral mucosa; Cell type: squamous epithelial cells; Level: Strong; Expression type: Staining; Reliability: Supportive |
|  | Tissue: ovary; Cell type: follicle cells; Level: Weak; Expression type: Staining; Reliability: Supportive |
|  | Tissue: ovary; Cell type: ovarian stroma cells; Level: Moderate; Expression type: Staining; Reliability: Supportive |
|  | Tissue: pancreas; Cell type: exocrine glandular cells; Level: Moderate; Expression type: Staining; Reliability: Supportive |
|  | Tissue: placenta; Cell type: decidual cells; Level: Strong; Expression type: Staining; Reliability: Supportive |
|  | Tissue: placenta; Cell type: trophoblastic cells; Level: Strong; Expression type: Staining; Reliability: Supportive |
|  | Tissue: prostate; Cell type: glandular cells; Level: Weak; Expression type: Staining; Reliability: Supportive |
|  | Tissue: salivary gland; Cell type: glandular cells; Level: Weak; Expression type: Staining; Reliability: Supportive |
|  | Tissue: seminal vesicle; Cell type: glandular cells; Level: Negative; Expression type: Staining; Reliability: Supportive |
|  | Tissue: skeletal muscle; Cell type: myocytes; Level: Moderate; Expression type: Staining; Reliability: Supportive |
|  | Tissue: skin; Cell type: epidermal cells; Level: Weak; Expression type: Staining; Reliability: Supportive |
|  | Tissue: small intestine; Cell type: glandular cells; Level: Weak; Expression type: Staining; Reliability: Supportive |
|  | Tissue: smooth muscle; Cell type: smooth muscle cells; Level: Moderate; Expression type: Staining; Reliability: Supportive |
|  | Tissue: spleen; Cell type: cells in red pulp; Level: Weak; Expression type: Staining; Reliability: Supportive |
|  | Tissue: spleen; Cell type: cells in white pulp; Level: Negative; Expression type: Staining; Reliability: Supportive |
|  | Tissue: stomach, lower; Cell type: glandular cells; Level: Moderate; Expression type: Staining; Reliability: Supportive |
|  | Tissue: stomach, upper; Cell type: glandular cells; Level: Moderate; Expression type: Staining; Reliability: Supportive |
|  | Tissue: testis; Cell type: cells in seminiferus ducts; Level: Weak; Expression type: Staining; Reliability: Supportive |
|  | Tissue: testis; Cell type: Leydig cells; Level: Weak; Expression type: Staining; Reliability: Supportive |
|  | Tissue: thyroid gland; Cell type: glandular cells; Level: Strong; Expression type: Staining; Reliability: Supportive |
|  | Tissue: tonsil; Cell type: germinal center cells; Level: Weak; Expression type: Staining; Reliability: Supportive |
|  | Tissue: tonsil; Cell type: non-germinal center cells; Level: Weak; Expression type: Staining; Reliability: Supportive |
|  | Tissue: tonsil; Cell type: squamous epithelial cells; Level: Strong; Expression type: Staining; Reliability: Supportive |
|  | Tissue: uterus, post-menopause; Cell type: cells in endometrial stroma; Level: Weak; Expression type: Staining; Reliability: Supportive |
|  | Tissue: uterus, post-menopause; Cell type: glandular cells; Level: Moderate; Expression type: Staining; Reliability: Supportive |
|  | Tissue: uterus, pre-menopause; Cell type: cells in endometrial stroma; Level: Negative; Expression type: Staining; Reliability: Supportive |
|  | Tissue: uterus, pre-menopause; Cell type: glandular cells; Level: Weak; Expression type: Staining; Reliability: Supportive |
|  | Tissue: vagina; Cell type: squamous epithelial cells; Level: Strong; Expression type: Staining; Reliability: Supportive |
|  | Tissue: vulva/anal skin; Cell type: epidermal cells; Level: Moderate; Expression type: Staining; Reliability: Supportive |

---
